# Elastoplastic Analysis of Circular Steel Tube of CFT Stub Columns under Axial Compression

**DOI:** 10.3390/ma15228275

**Published:** 2022-11-21

**Authors:** Hua Zhao, Rui Han, Weiguang Yuan, Shichun Zhao, Yuping Sun

**Affiliations:** 1 State Key Laboratory of Geohazard Prevention and Geoenvironment Protection, Chengdu University of Technology, Chengdu 610059, China; 2 Department of Civil Engineering, Sichuan University, Chengdu 610207, China; 3 School of Civil Engineering, Southwest Jiaotong University, Chengdu 610031, China; 4 Department of Architecture, Graduate School of Engineering, Kobe University, Kobe 657-8501, Japan

**Keywords:** CFT, cold-formed circular steel tube, stub column, biaxial stress state, local buckling

## Abstract

Composite action between the components of the concrete-filled steel tube (CFT) is complex and it is difficult to accurately obtain the experimental relationship between the steel tube and the core concrete of CFT columns. The triaxially stressed core concrete has been studied by hydrostatic test in past research, while little research has been focused on the mechanical behavior of steel tube of CFT columns. It is difficult to obtain the experimental constitutive relationship of the steel tube of CFT columns to reflect the real-time influence of biaxial stress state and local buckling of steel plate on the steel tube. To clarify the mechanical behavior of the steel tube of CFT columns, this paper proposed an elastoplastic analytical method considering biaxial stress state and local buckling of steel tube to obtain the stress–strain curve of the steel tube. This method applied the Hook’s law and the plasticity theory to interpret the information conveyed by the measured vertical and hoop strain histories of the steel tube. To verify its effectiveness, 11 circular concrete-filled steel tube stub columns were fabricated and tested under axial compression. Superposition results of the axial load–strain of steel tube and core concrete were compared against the experimental curves. The widely used Sakino–Sun model of the confined concrete was adopted to calculate the axial load–strain curve of the core concrete. Satisfactory agreements between the calculated and experimental results confirmed the rationality of the proposed method in tracing the constitutive relation of the biaxially stressed steel tube even after the occurrence of the local buckling. The obtained stress–strain relationship is critical for establishment of mathematical constitutive model and finite element model of steel tube.

## 1. Introduction

Structurally benefitting from the composite action, concrete-filled steel tube (CFT) columns with enhanced strength and ductility have been increasingly widespread as gravity-sustaining components especially in high-rising buildings. It is recognized that the structural benefits of CFT are to be achieved by the effective confinement provided by the steel tube with a certain range of outer diameter-to-thickness ratio *D*/*t* to obtain the core concrete triaxially stressed and avoid the spalling of shell concrete. Moreover, the infilled concrete prevents the inward local bulking of the steel tube with improved stability, especially for the thin-walled steel tube. However, the biaxial stress state of steel tube resulting from the concrete dilation may conversely lead to a strength reduction in the steel tube. It is quite complex and difficult to quantify the influence of the composite action to evaluate the mechanical behavior of CFT. For decades, considerable research has been trying to develop a deep understanding of the axial mechanical behavior of CFT columns, but much emphasis has been only placed on the influence of variables on the overall performance of CFT, such as loading conditions [1,2,3], slenderness [2,4,5,6], concrete strength [3,4,5,6,7,8], the yield stress of steel tube [4,5], *D*/*t* ratio [4,7,8,9,10], the bond strength [8,11], stainless steel [12], recycled aggregate concrete [12], and section type [13,14,15].

To solve this problem, a common strategy is the method of superposition, which requires individual investigation into the performance of confined concrete and steel tube. As for the confined concrete, several triaxial pressure tests covering a wide range of concrete strength and confining stress have been conducted to comprehend its mechanical behavior [16,17,18,19,20]. A variety of stress–strain models are also available for the confined concrete, such as the trilinear model of Chan (1995), the bilinear model of Roy et al. (1964), the three-branch curve of Soliman et al. (1967) and Kent et al. (1971), the four-stage model of Park et al. (1974) and Sheikh et al. (1980), and the continuous model of Sargin (1971) and Mander et al. (1988) [21]. In 1994, as a part of the fifth phase of the U.S.–Japan Cooperative Earthquake Research Program, a unified stress–strain model was proposed by Sakino and Sun for the confined concrete by conventional transverse reinforcements or/and steel tube [22,23,24]. More recently, modified models of confined concrete have been proposed on the basis of these classical models [25,26,27,28,29].

However, limited attention has been received to clarify the mechanical behavior of the steel tube of CFT and it is difficult to obtain the stress–strain curve of the steel tube through experiments on CFT. At present, two indirect strategies have been applied in the existing literature to obtain the stress–strain curve of the steel tube of CFT. One method is the experimental investigation on the hollow steel tube (CHS) [30,31,32,33]. It can reflect the mechanical behavior of axially stressed CHS, but the effect produced by the core concrete on the behavior of the steel tube has been ignored by this means. In another method, the plastic theory was applied to conduct elastoplastic analysis on CFT. Incremental Prandtl–Reuss theory as the most commonly used type of the plastic theory was verified reliable to obtain the stress–strain relation of the steel tube of CFT [34,35]. However, it was found that the stresses were sensitive to the strains and a minor inaccuracy in predicting the strains of steel tube could lead to a huge error in stress prediction by this theory, and the convergence problem of the iterative calculation procedure also made it difficult for practical application [36]. So, it is urgent to develop a rational and simple approach to obtain the experimental stress–strain curve of the steel tube of CFT to comprehend its mechanical behavior. Based on the obtained experimental stress–strain curve of the steel tube of CFT, the mathematic axial stress–strain model of steel tube of CFT is anticipated to be built to consider the biaxial stress state and local buckling, which are not clearly considered or just considered based on simulation results in current constitutive model of steel tube infilled with concrete.

The plastic theory in form of deformation type is a potential solution to solve the above problems. Compared with the incremental Prandtl–Reuss theory, it is mathematically less complex and easier for implementation based on certain assumptions. In the past decades, the deformation theory of plasticity was widely used to estimate the buckling behavior of axially compressed CHS [37], axially and biaxially stressed steel plates under proportional and non-proportional loading [38,39], and circular stainless steel tube under combined internal pressure and axial compression [40]. However, the application of this theory as an alternative in the field of CFT is rare and needs to be investigated. Li et al. adopted this theory to calculate the biaxial stress of the stainless steel tube of circular concrete-filled stainless steel tube (CFSST) stub columns with appropriate consideration of the tube buckling and biaxial stress condition [36], but the material characteristics of the stainless steel tube differs from the steel tube. Grigoryan et al. presented a method to determinate the ultimate axial load of CFT based on this theory [41], but the constitutive law of the steel tube needs to be further investigated.

The stress–strain relationship of circular steel tube infilled with concrete is essential to clarify the mechanical behavior of CFT columns, but it is difficult to obtain the stress–strain relationship of steel tube through existing analytical methods. This paper proposed a method to obtain the constitutive curve of the circular steel tube infilled with concrete by applying the classical Hook’s law and the deformation theory of plasticity to analyze the measured vertical and hoop strains of the steel tube based on experiments on 11 CFT stub columns. Compared with existing method to obtain the full-range stress–strain relationship of steel tube of CFT columns, the proposed method considers the local buckling and the influence of infilled concrete on the steel tube with less mathematically complex for implementation. The experimental axial stress–strain curves of steel tube of the specimens were obtained using the proposed method. The effects of local bulking and concrete dilation on the performance of the steel tube of CFT are well-reflected. Finally, the accuracy of the proposed method was verified by comparing with the experimental curve.

## 2. Experimental Program

### 2.1. Details of the Specimens

**Figure 1:**
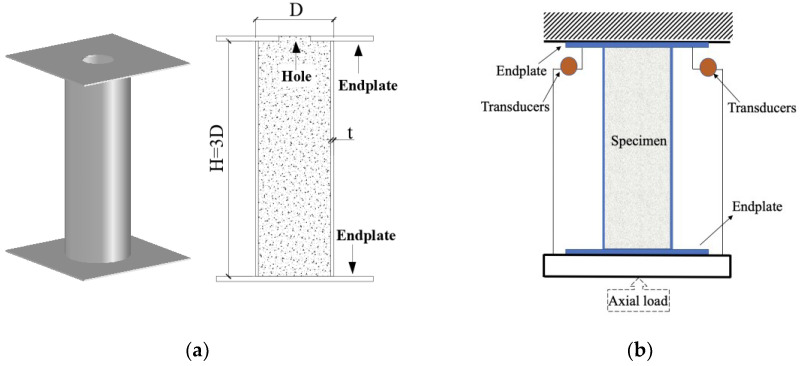
The specimens and test setup: (**a**) the specimens; (**b**) the test setup.

A total of 11 CFT stub columns were constructed and tested under axial compression as shown in Figure 1a. All specimens were fabricated with height-to-outer diameter *H*/*D* of 3 to avoid the end effects. A steel plate with the yield stress *f_sy_* of 305 MPa, 312 MPa and 329 MPa was cold-formed into a circular section with the outer diameter-to-thickness ratio *D*/*t* ranging from 55 to 115. The steel tube with *D*/*t* of 31 was seamless steel tube. The generalized diameter-to-thickness ratio α (= *Df_sy_*/*tE*_s_ [42,43]) of the steel tube, which is similar to the slenderness ratio in AISC and well-used in Japan to measure the local buckling strength of CHS, was covering 0.06 to 0.17. *E*_s_ is the Young’s Modulus of the steel. Two endplates were welded to the top and toe of the steel tube to ensure the cross-section of the specimens could simultaneously sustain the force.

**Figure 2:**
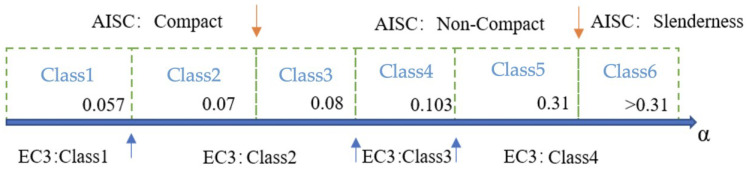
Section classification of steel tube [45].

The concrete was cast in the vertical position through the hole of the top endplate and progressive vibration was applied to eliminate air pockets and guarantee a homogeneous mix. During the cast, the concrete was filled spilling over the top endplate to guarantee the amount of concrete was enough to fill the air pockets. After the overflow of concrete sank into the steel tube to fill the air pockets, the hole was emptied out and filled with water 8 h after casting to provide a humid curing condition. Before the loading test, high-strength mortar was squeezed into the steel tube through the hole of top endplate to fill the gap caused by shrinkage of concrete and make the concrete surface flush with the top endplate. Meanwhile, the corresponding standard concrete cubes with a dimension of 150 mm × 150 mm × 150 mm [44] were cast for the concrete strength test. On the test day, the compressive strength *f_cu,k_* of the standard concrete cubes were 58 MPa, 70 MPa, and 93 MPa, respectively, for three grades of concrete. To convert the compressive strength *f_cu,k_* of the concrete cube to corresponding concrete cylinder (100 mm × 200 mm) strength *f_cu,kcy_*, the Equation (1) was adopted according to the reference [35]. The detailed parameters of the specimens are listed in Table 1. The section type of steel tube was classified according to the AISC and EC3 codes as shown in Figure 2 [45], in which the upper limits of the α were specified as 0.11 and 0.103, respectively.

(1) $$f_{cu,kcy}=\frac{0.8513f_{cu,k}-1.5998}{0.96}$$

### 2.2. Test Apparatus and Instrumentations

The specimens were tested under concentric axial compression by a servo-controlled hydraulic machine. The load was imposed in small increments at an initial rate of 2 kN/s. After the load development was steady, the rate was increased and maintained a constant rate of 5 kN/s until the applied load was about 80% of the predicted maximum load [46]. Then, the loading was controlled at the rate of 0.017 mm/s at the final stage of the test. The test setup is shown in Figure 1b.

**Figure 3:**
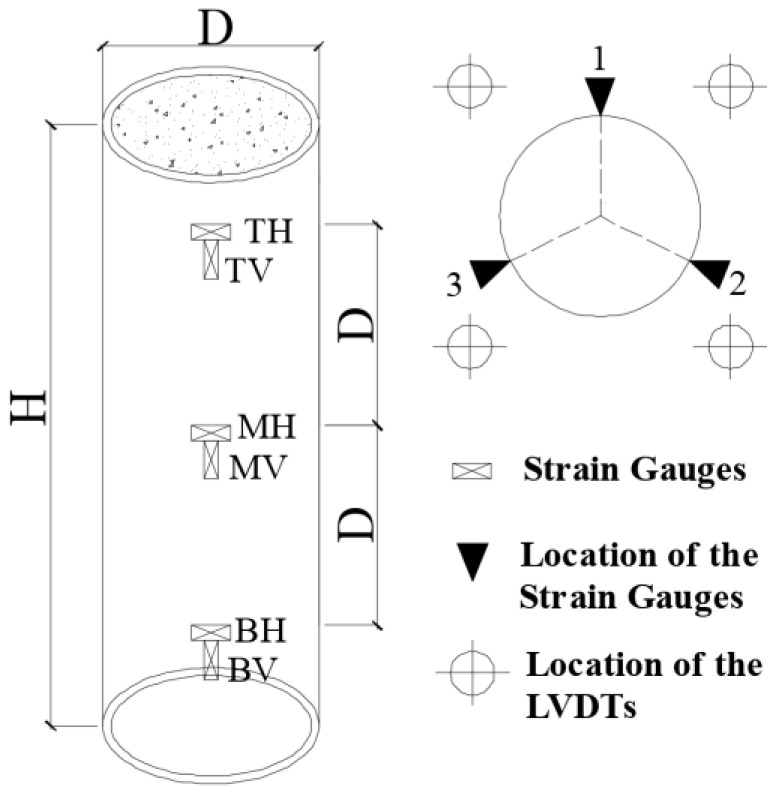
The location of the strain gauges and LVDTs.

Four displacement transducers (LVDTs) were instrumented at symmetric locations to record the axial displacement of the specimens. Paired vertical and horizontal strain gauges were arranged on the exterior surface of the steel tube from top to toe to monitor and record the vertical deformation and the perimeter expansion of the steel tube. At the early stage of loading, the data from LVDTs and strain gauges was also used to ensure the compressive loading was applied evenly without eccentricity. The location of the strain gauges and LVDTs is illustrated in Figure 3.

## 3. Test results and Discussions

**Figure 4:**
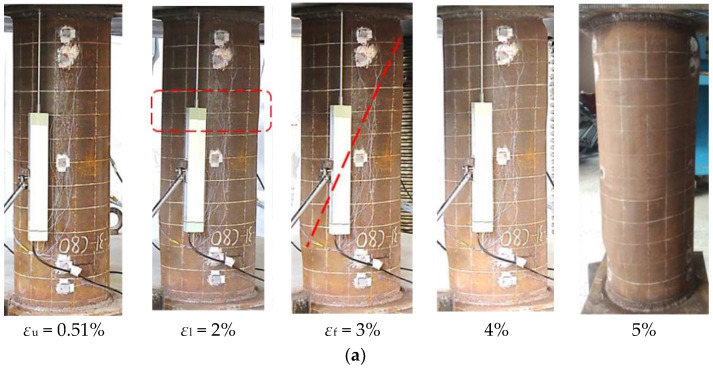

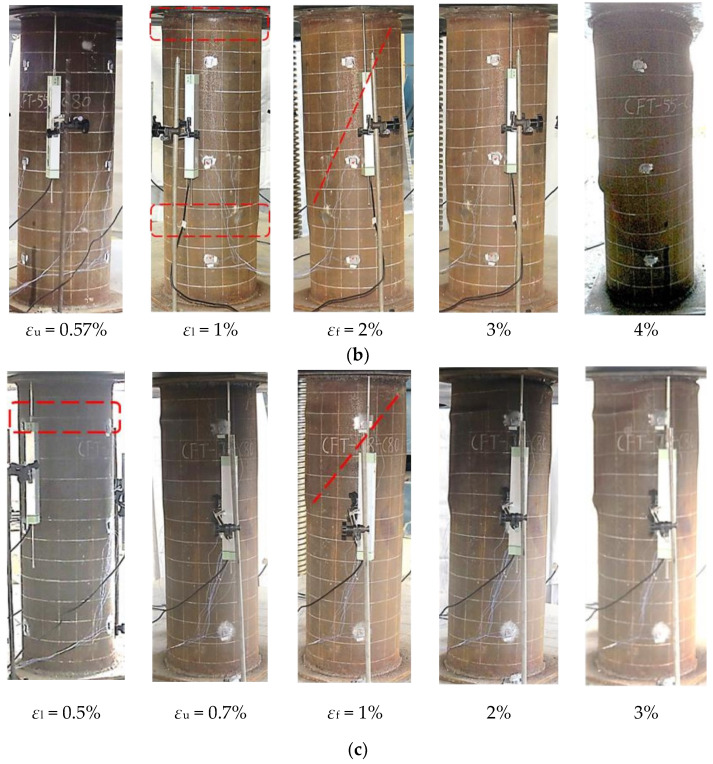
Deformation development: (**a**) Class 2: CFT-31-C80; (**b**) Class 4: CFT-55-C80; (**c**) Class 5: CFT-78-C80.

Typical deformation development of the specimens is shown in Figure 4, which takes the CFT-31-C80, CFT-55-C80, and CFT-78-C80 as representative examples for specimens classified in Class 2, Class 4, and Class 5 series. *Ε*_l_, *ε*_f_, and *ε*_u_ are the strains corresponding to onset of the local buckling of the steel tube, formation of the concrete crushing shear plane, and the ultimate strength of the specimens, respectively. These specific strains are chosen in Figure 4 because the test CFT columns at these strains exhibited specific experimental phenomenon and load change.

As shown in Figure 4, expansion of steel tube was initialized within the region near the endplates and the middle height of the specimens. At the strain of 2%, bulge of the steel tube of CFT-31-C80 was founded close to the middle height of the column after the ultimate strength at strain of 0.51%. Then the concrete was successively crushed at strain of 3%. The local buckling of CFT-55-C80 was found at strain of 1% near the top endplate and middle region of the steel tube after the ultimate strength at strain of 0.57%, and the shear plane of crushed concrete was formed at strain of 2%. Different from the specimens of Class 2 and Class 4, local buckling of the CFT-78-C80 at strain of 0.3% was prior to the ultimate strength at strain of 0.7%, and the shear crushing of concrete occurred at a higher height of the column. It should be noted that the shear failed concrete caused secondary local buckling of the steel tube, which dominated buckle deformation of the steel tube with increasing axial compression.

**Figure 5:**
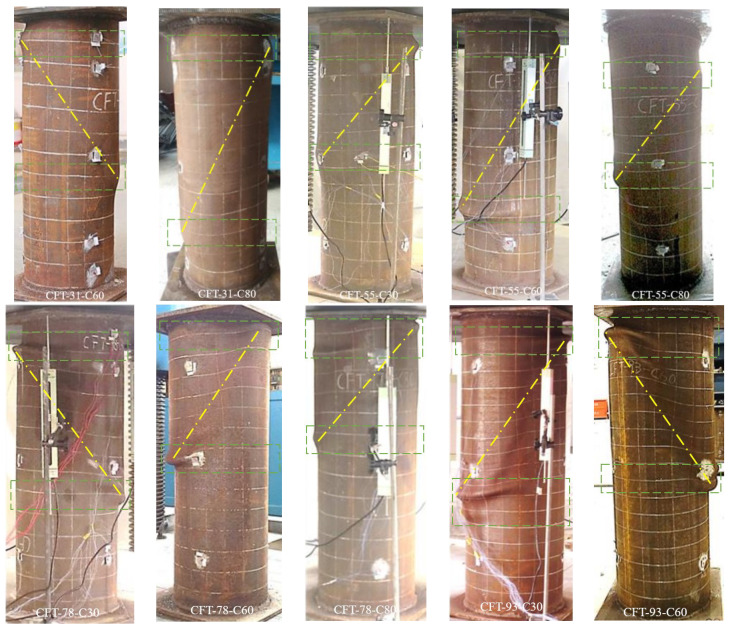
Failure modes of the specimens.

Crushing of concrete in shear failure and local buckling of the steel tube were observed in all the specimens. Figure 5 shows the failure mode of the all the specimens except for CFT-125-C30, of which the test was terminated in advance due to the fluctuated hydraulic pressure of the loading devices. The dash line and dash-dot line locate the local bucking of steel tube and the shear failed concrete, respectively. With increasing *D*/*t*, wrinkles of the buckled steel tube were severer.

**Figure 6:**
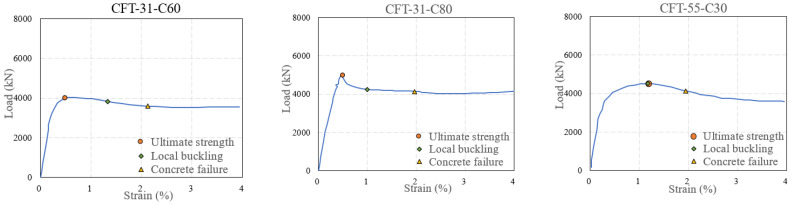

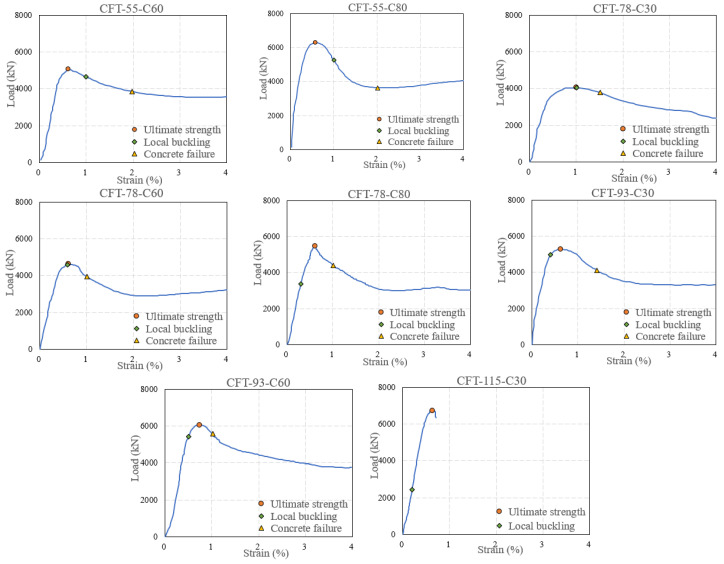
Experimental load–strain curve of the specimens.

Figure 6 presents the measured load–strain curve of the specimens. The strain was obtained by dividing the average measured results of the four LVDTs by the height *H* of the specimens. Symbols of “○”, “◇”, and “△” marked the ultimate strength of the specimens, local buckling of the steel tube, and the concrete failure, respectively. Local buckling of steel tube was prior to the ultimate strength when the thickness of steel tube exceeding the limitation of Class 4, which indicated that the influence of local buckling was greater on the columns with a thinner-walled steel tube. For specimens with *D*/*t* of 31, 55, and 78, the local buckling was delayed with decreased concrete strength. Concrete shear failure of specimens in Class 2 and Class 4 occurred around strain of 2%, and the shear failure was advanced with increasing *D*/*t*. Higher strength concrete of specimens showed earlier shear failure due to less ductility. The residual load capacity of specimens in Class 2 and Class 4 at strain of 4% was around 4000 kN, which was higher than that of specimens in Class 5. With increasing concrete strength and ratio of *D*/*t*, the strength degradation of the columns was steeper.

**Figure 7:**
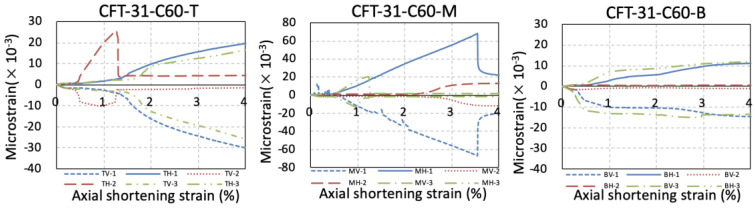

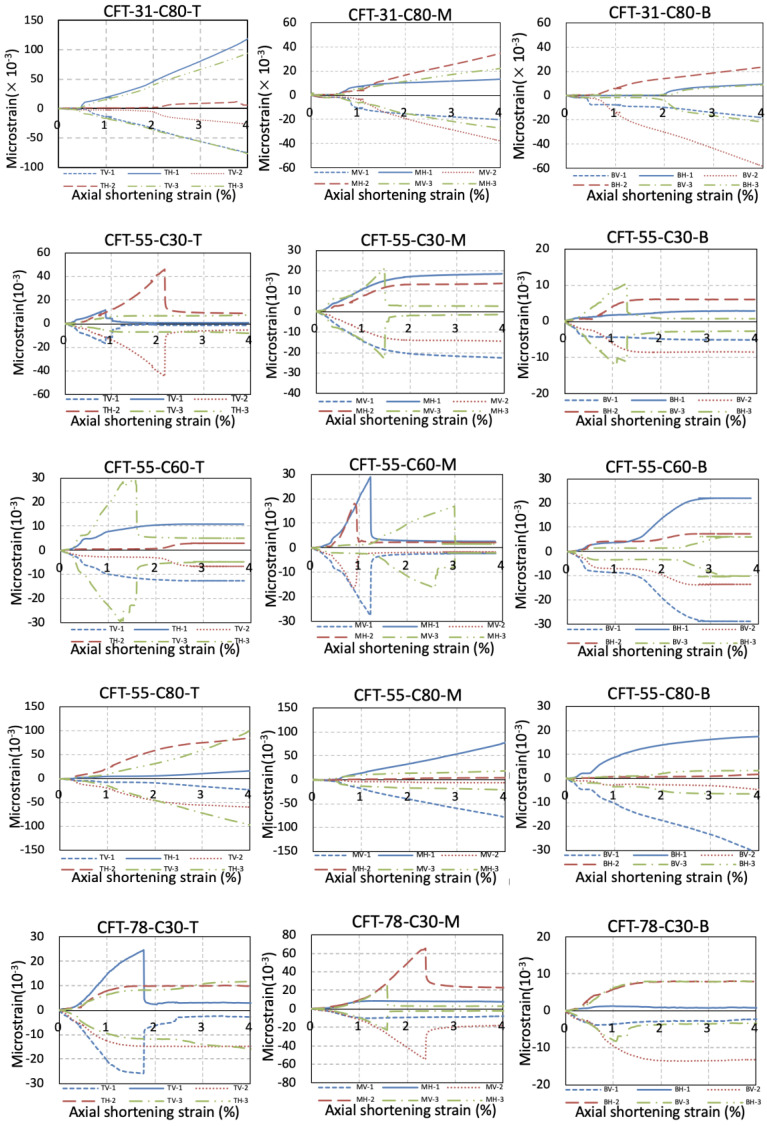

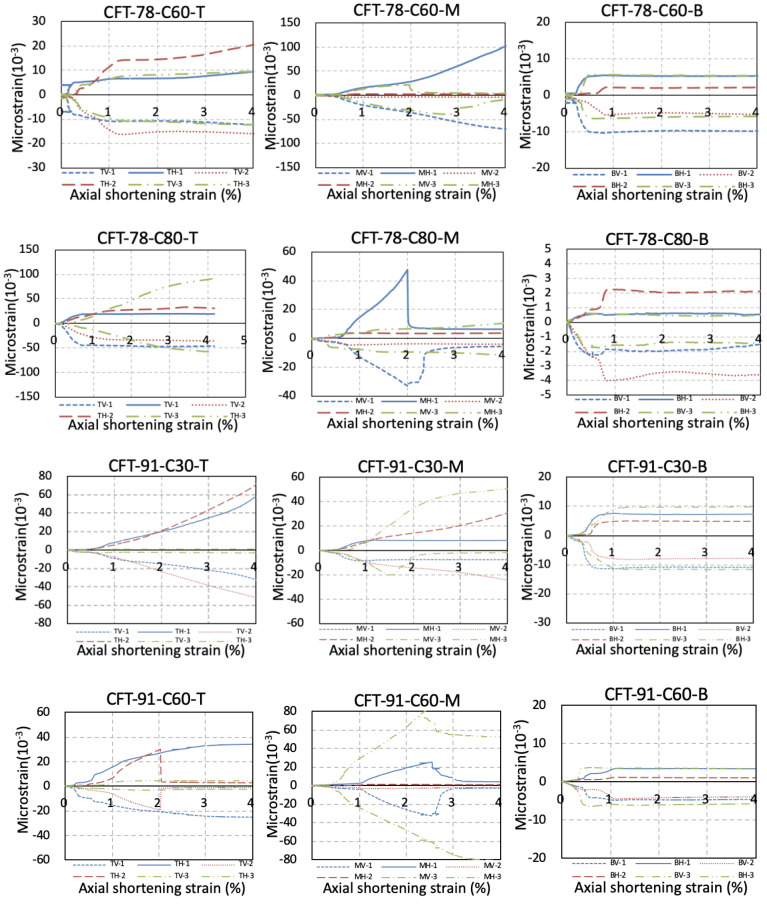
The measured strain of the steel tube.

As shown in Figure 7, paired vertical and hoop strain histories of 9 points on the external surface of the steel tube of the specimens were measured. In this figure, the T, M, and B represented the strain measured at the top, middle, and bottom height of the column, respectively. The V and H represented the vertical and hoop strain, respectively. The numbers 1, 2, and 3 indicated the different positions of the measured points distributing across the section of the specimens as shown in Figure 2. The measured strain of steel tube was smaller at lower height of the column due to the stress delivery through bond strength between the steel tube and core concrete.

## 4. Theoretical Relation of the Axial Stress–Strain between CHS and Steel Tube of CFT

It is difficult to obtain the experimental stress–strain curve of the steel tube directly from the test on the CFT, and only the load-displacement curve of the specimens and strain history of the steel tube can be directly measured in the test. Therefore, this paper is intended to propose an alternative method to obtain the experimental constitutive curve of the biaxially stressed steel tube of the specimens. The concept of this method is to establish a relationship of the axial stress–strain between CHS and steel tube of CFT by using the deformation theory of plasticity to analyze the measured vertical and hoop strains of the steel tube of CFT under the assumption of proportional loading of the steel tube. Once the experimental relationship of the axial stress–strain curve between CHS and steel tube of CFT is known, the axial experimental stress–strain curve of the steel tube of CFT can be converted from the stress–strain curve of CHS, which can be obtained by experiments on CHS or calculated by the existing effective constitutive model.

**Figure 8:**
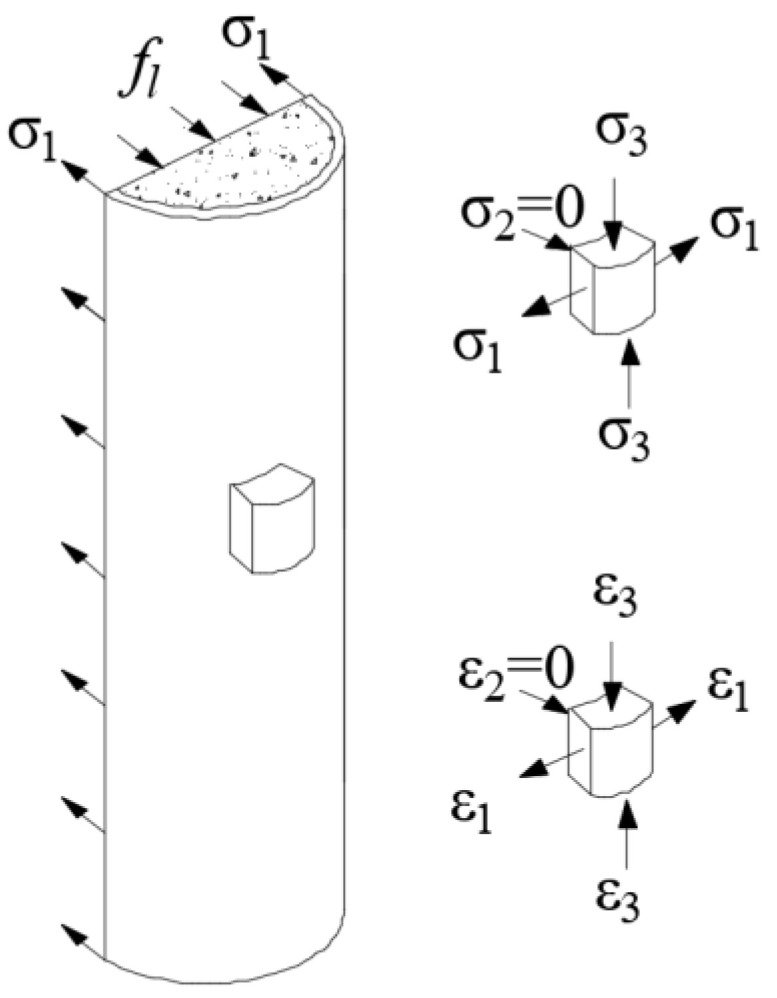
Stress components of steel tube.

Based on the deformation theory of plasticity, a transformation relationship between the biaxial stress–strain curve and the axial stress–strain curve was derived, which essentially follows the content of the literatures [47,48,49]. The cubic element of steel is assumed to be subject to principal stress *σ*_1_, *σ*_2_ = 0, and *σ*_3_ with corresponding strain *ε*_1_, *ε*_2_ = 0, and *ε*_3_ as shown in Figure 8. The *σ*_1_ and *σ*_3_ are correspondent to the hoop and axial stress of the steel tube of CFT, respectively. At the elastic stage, the principal stress and strain follow the Hook’s law as Equations (2)–(4), and the Poisson’s ratio *ν* is assumed to be 0.3 at this phase.

(2) $$\varepsilon_{1}=\frac{1}{E}\left[\sigma_{1}-\nu \left(\sigma_{2}+\sigma_{3}\right)\right]$$

(3) $$\varepsilon_{2}=\frac{1}{E}\left[\sigma_{2}-\nu \left(\sigma_{1}+\sigma_{3}\right)\right]$$

(4) $$\varepsilon_{3}=\frac{1}{E}\left[\sigma_{3}-\nu \left(\sigma_{1}+\sigma_{2}\right)\right]$$

At the plastic stage, the relationship between octahedral shear stress *τ_oct_* and the equivalent stress $\bar{\sigma }$ can be expressed by Equations (5) and (6) according to the theory of plasticity.

(5) $$\tau_{oct}=\frac{1}{3}\sqrt{{\left(\sigma_{1}-\sigma_{2}\;\right)}^{2}+{\left(\sigma_{2}-\sigma_{3}\;\right)}^{2}+{\left(\sigma_{1}-\sigma_{3}\;\right)}^{2}}$$

(6) $$\tau_{oct}=\frac{\sqrt{2}}{3}\bar{\sigma }$$

For simplicity, *α*_1_, *α*_2,_ and *α*_3_ are ratios of principle stresses defined by Equation (7) and the *τ_oct_* can be simplified as Equation (8).

(7) $$\alpha_{1}=\sigma_{1}/\sigma_{3},\alpha_{2}=\sigma_{2}/\sigma_{3},\alpha_{3}=\sigma_{3}/\sigma_{3}$$

(8) $$\tau_{oct}=\frac{\sqrt{2}}{3}\sigma_{3}\sqrt{\alpha_{1}{}^{2}+\alpha_{2}{}^{2}+1-\alpha_{1}\alpha_{2}-\alpha_{1}-\alpha_{2}}$$

Octahedral shear stress *τ*_oct,a_ in axial stress state (*α*_1_ = 0, *α*_2_ = 0, *α*_3_ = 1) and octahedral shear stress *τ*_oct,b_ in biaxial stress state (*α*_2_ = 0, *α*_3_ = 1) can be obtained by Equation (9) and Equation (10), respectively. The *σ*_3*a*_ and *σ*_3*b*_ are the axial stress of steel under axial and biaxial stress state, respectively.

(9) $$\tau_{\mathrm{oct},a}=\frac{\sqrt{2}}{3}\sigma_{3}=\frac{\sqrt{2}}{3}\sigma_{3a}$$

(10) $$\tau_{\mathrm{oct},b}=\frac{\sqrt{2}}{3}\sigma_{3}=\frac{\sqrt{2}}{3}\sigma_{3b}\sqrt{\alpha_{1}{}^{2}+1-\alpha_{1}}$$

**Figure 9:**
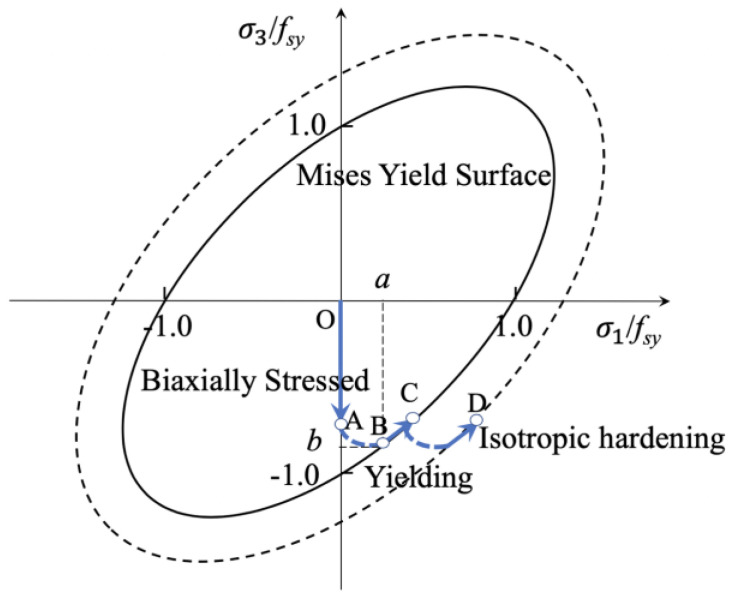
Yield criteria and flow rules of steel tube.

If Von Mises criterion is assumed to be valid as shown in Figure 9, octahedral shear stress *τ_oct_* is independent of stress state and only related to the principal stress. That is, equivalent stress determines the yield of steel no matter in what stress state as expressed in Equation (11). The Von Mises criterion for the biaxially stressed steel tube can be expressed as Equation (12).

(11) $$\tau_{\mathrm{oct},a}=\tau_{\mathrm{oct},b}$$

(12) $$\sigma_{1}^{2}-\sigma_{1}\sigma_{3}+\sigma_{3}^{2}=f_{sy}^{2}$$

Thus, the theoretical relationship of the axial stress of steel in the axial and biaxial conditions is obtained by Equation (13).

(13) $$\sigma_{3b}=\sigma_{3a}/\sqrt{\alpha_{1}{}^{2}+1-\alpha_{1}}$$

The reduction factor of the axial stress of steel from axial to biaxial stress state is defined as *ψ* by Equation (14) to calculate the difference of axial stress–strain curve between the biaxially stressed steel tube of CFT and the corresponding axially stressed CHS. Because the hoop and axial stress of the steel tube of CFT cannot be measured directly from experiments on CFT, the calculation method of *α*_1_ is to be presented in the following part to obtain the reduction factor *ψ*.

(14) $$\psi =1/\sqrt{\alpha_{1}{}^{2}+1-\alpha_{1}}$$

Based on the assumption that the components of deviatoric stress and strain are proportional, Equation (15) can be obtained. *μ*′ is the function of the strain state.

(15) $$\frac{\sigma_{1}-\sigma_{2}}{\varepsilon_{1}-\varepsilon_{2}}=\frac{\sigma_{2}-\sigma_{3}}{\varepsilon_{2}-\varepsilon_{3}}=\frac{\sigma_{3}-\sigma_{1}}{\varepsilon_{3}-\varepsilon_{1}}=2\mu^{\prime }$$

If the assumptions that (1) the equivalent stress $\bar{\sigma }$ is a function of the equivalent strain $\bar{\varepsilon }$ as Equation (16), and (2) the volume of steel is incompressible (Poisson’s ratio *ν* = 0.5) are valid, the *μ*′ can be calculated by Equation (17). *E*′ is the function of the equivalent strain $\bar{\varepsilon }$.

(16) $$\bar{\sigma }=E^{\prime }\bar{\varepsilon }$$

(17) $$\mu^{\prime }=\frac{E^{\prime }}{2\left(1+\upsilon \right)}=\frac{E^{\prime }}{3}$$

Combining Equations (15) and (17), the relationship of the principal stress and strain can be obtained as Equations (18)–(20).

(18) $$\varepsilon_{1}=\frac{1}{E^{\prime }}\left[\sigma_{1}-\frac{1}{2}\left(\sigma_{2}+\sigma_{3}\right)\right]$$

(19) $$\varepsilon_{2}=\frac{1}{E^{\prime }}\left[\sigma_{2}-\frac{1}{2}\left(\sigma_{1}+\sigma_{3}\right)\right]$$

(20) $$\varepsilon_{3}=\frac{1}{E^{\prime }}\left[\sigma_{3}-\frac{1}{2}\left(\sigma_{1}+\sigma_{2}\right)\right]$$

For simplicity, *β*_1_, *β*_2_, and *β*_3_ are the ratios of principal strain defined by Equation (21) and *β*_2_ = 0 in the biaxial stress state. For the steel tube of CFT, *β*_1_ is the ratio of axial strain to hoop strain.

(21) $$\beta_{1}=\varepsilon_{1}/\varepsilon_{3},\beta_{2}=\varepsilon_{2}/\varepsilon_{3},\beta_{3}=\varepsilon_{3}/\varepsilon_{3}$$

Substituting Equations (7) and (21) into Equations (2)–(4) and Equations (18)–(20), the *α*_1_ at the elastic and plastic stage can be calculated by Equation (22).

(22) $$\alpha_{1}=\left\{\begin{array}{cc} \frac{\beta_{1}+0.3}{1+0.3\beta_{1}} & Elastic\;stage\\ \frac{\beta_{1}+0.5}{1+0.5\beta_{1}} & Plastic\;stage \end{array}\right.$$

## 5. Analytical Results

**Figure 10:**
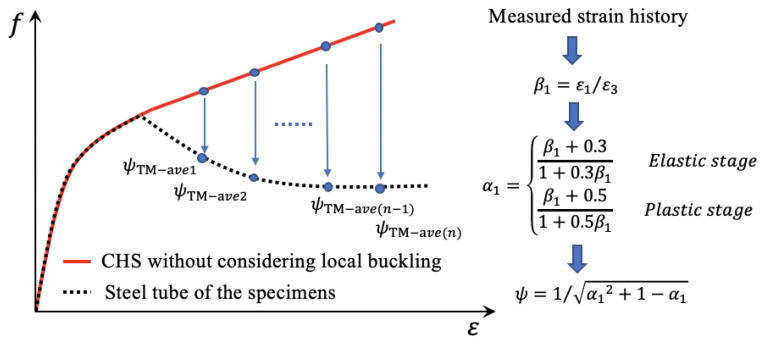
Calculation flow of the proposed method.

To obtain the axial stress–strain curve of the steel tube of the specimens, the experimental real-time *β*_1_ was substituted into Equation (4) to obtain α1 and then the reduction factor *ψ* was calculated by substituting the α1 into Equation (3). Because *β*_1_ was an experimental result, the reduction factor *ψ* determined by *β*_1_ was comprehensive to reflect the effect of concrete dilation, bond strength, and local buckling on the performance of the steel tube. Moreover, the stress–strain curve of CHS was obtained by the constitutive model of CHS stub columns without considering the local buckling of CHS to avoid duplication of consideration with the reduction factor *ψ*. The concept and calculation flow of this proposed method are shown in Figure 10. The reduction factor *ψ*_TM-ave_ is the averaged *ψ* within the top and middle region of the steel tube, which is defined and used in Section 5.2.

### 5.1. Ratios β1 of Hoop Strain to the Vertical Strain of the Measured Point

**Figure 11:**
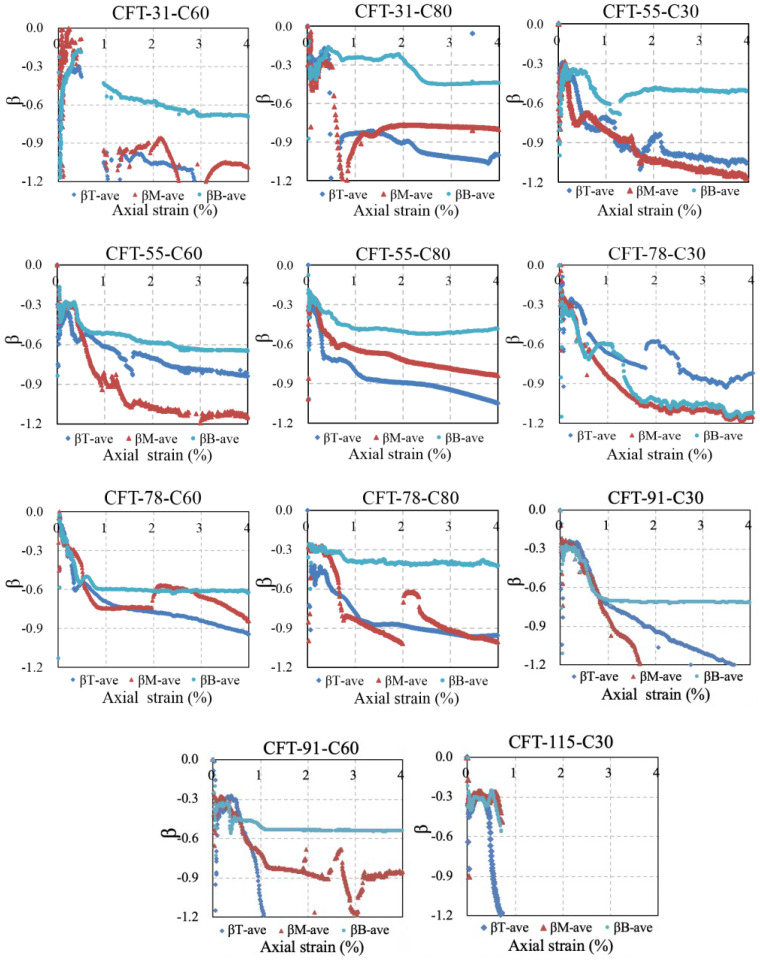
Ratio of hoop strain to vertical strain β1.

Figure 11 illustrated the ratio *β*_1_ of the hoop strain to the vertical strain of the measured points. Based on the assumption of plane-remain-plane, *β*_T-ave_, *β*_M-ave_, and *β*_B-ave_ were obtained by averaging the measured *β*_1_ of three points across the section of the steel tube from the top to the bottom of the specimens. The Poisson’s ratio of steel tube was around 0.3 at the initial stage as shown in Figure 11. With increasing axial strain, almost all the ratios *β*_T-ave_ and *β*_M-ave_ of the steel tube within the upper region dropped faster than *β*_B-ave_ within the bottom region, which revealed that the dilation of core concrete or the local buckling of steel tube was more likely to develop within upper-region steel tube instead of the bottom part. This phenomenon has also been found in research [1,3]. The performance of the upper-region steel tube was of representative of the whole steel tube. So, the *ψ*_TM-ave_ of the upper region was chosen to calculate the stress–strain curve of the steel tube of the specimens.

### 5.2. Real-Time Reduction Factor ψ

**Figure 12:**
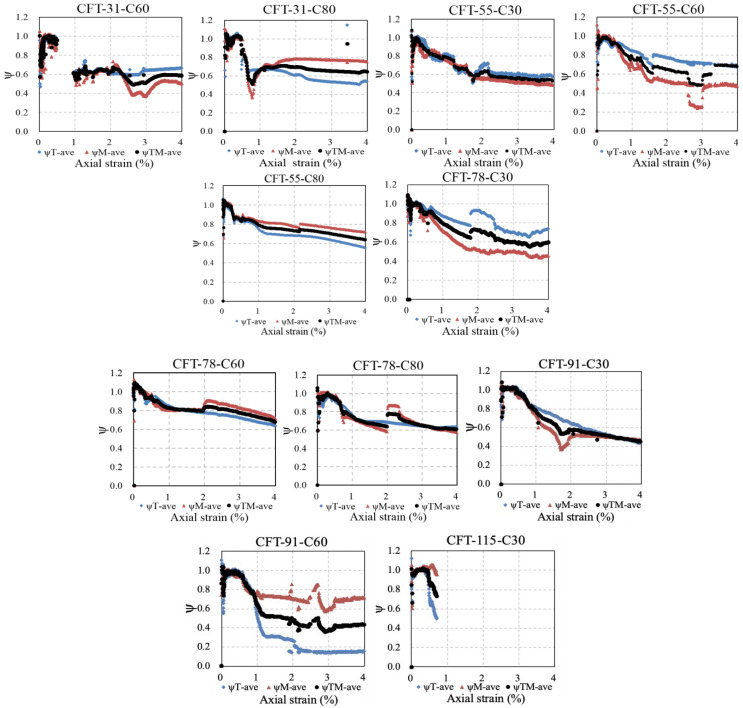
Reduction factor ψ.

The calculation results of the reduction factor *ψ* were illustrated in Figure 12. The symbols of rhombus and triangle represent the average reduction factors *ψ*_T-ave_ and *ψ*_M-ave_ of the top and middle region of the specimens, respectively, and the dotted line of *ψ*_TM-ave_ averaging *ψ*_T-ave_ and *ψ*_M-ave_ was employed to calculate the stress–strain curve of the steel tube of the specimens. It appeared that the reduction factors *ψ*_T-ave_, *ψ*_M-ave_, and *ψ*_TM-ave_ maintained around 1.0 at the initial stage, which exactly reflected the noninterference between the steel tube and core concrete at the elastic stage. Once the interaction between the concrete and steel tube occurred, the reduction factors *ψ*_T-ave_, *ψ*_M-ave_, and *ψ*_TM-ave_ decreased.

### 5.3. The Constitutive Model of CHS

**Figure 13:**
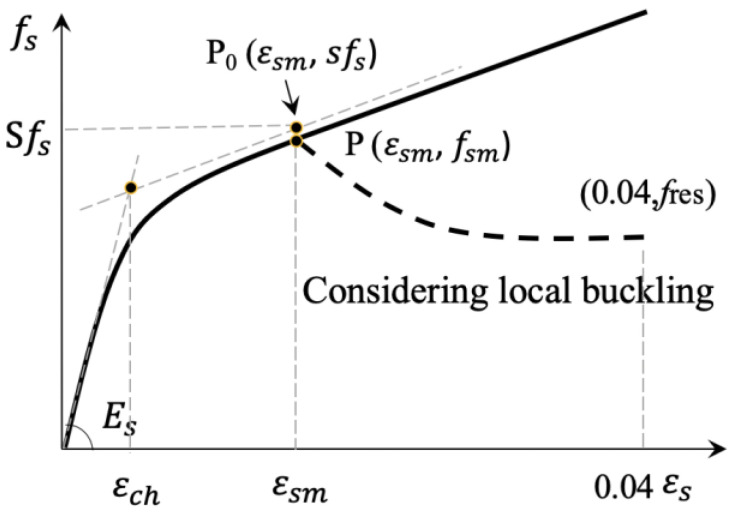
Constitutive model of CHS.

The constitutive model of CHS in reference [42] was adopted in this paper to derive the stress–strain curve of the steel tube of the specimens. As shown in Figure 13, the solid curve *f_chsl_*-*ε* was the model of CHS without considering the local buckling effect and the dashed line *f*_chs_-*ε* after point P was the descending branch to consider this effect. Point P indicates the local buckling of the CHS. The S represents the ratio of the measured local buckling strength to the measured yield strength *f_sy_* of CHS. The Q was the second stiffness ratio of the ascending portion of the CHS model. The *ε_ch_* was the characteristic strain. The *f_sm_* and *ε_sm_* were the peak stress and the strain corresponding to *f_sm_*. The *f_res_* was the residual stress of CHS at the compressive strain of 0.04. The mathematical expressions and parameters of the model were listed in Appendix A.

### 5.4. Stress–Strain Curves of the Steel Tube of the Specimens

The stress–strain curves *f_st_*-*ε* of the steel tube of the specimens were obtained by multiplying the curves *f_chsl_-ε* of the corresponding CHS by the reduction factors *ψ_TM-ave_* as Equation (23). The *f_st_* was correspondent to the absolute value of *σ*_3_.

(23) $$f_{st}\left(\varepsilon \right)=f_{chsl}\left(\varepsilon \right)\psi_{TM-ave}\left(\varepsilon \right)$$

**Figure 14:**
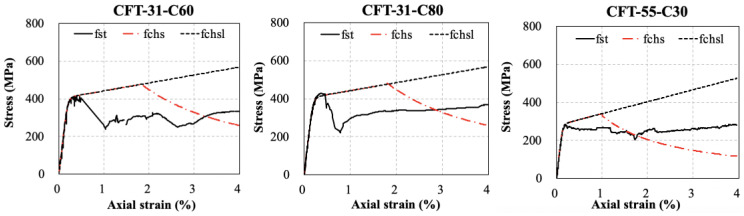

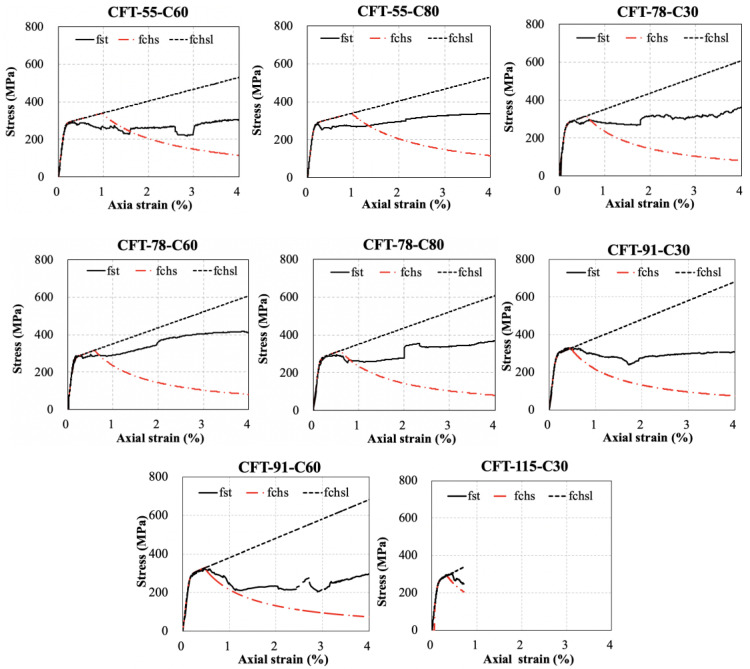
Calculated stress–strain curve of the steel tube of CFT and CHS.

The calculation results were shown in Figure 14. For comparisons, curves *f*_chs_-*ε* of the corresponding CHS considering the local buckling effect were also presented. It demonstrated that the strength degradation of the steel tube of CFT occurred earlier than that of CHS, which was caused by the biaxial stress condition of the steel tube of CFT. However, the degradation almost happened at the same time for both kinds of steel tubes with an increasing ratio of *D*/*t*, especially with the *D*/*t* exceeding 91. Moreover, the residual strength of the steel tube of CFT at the strain of 0.04 indeed improved due to the support of infilled concrete and with increasing *D*/*t*, the improvement became greater. Therefore, the stress–strain model for CHS indeed must be modified for the application of the steel tube of CFT to consider the influence produced by the core concrete.

## 6. Verification

**Figure 15:**
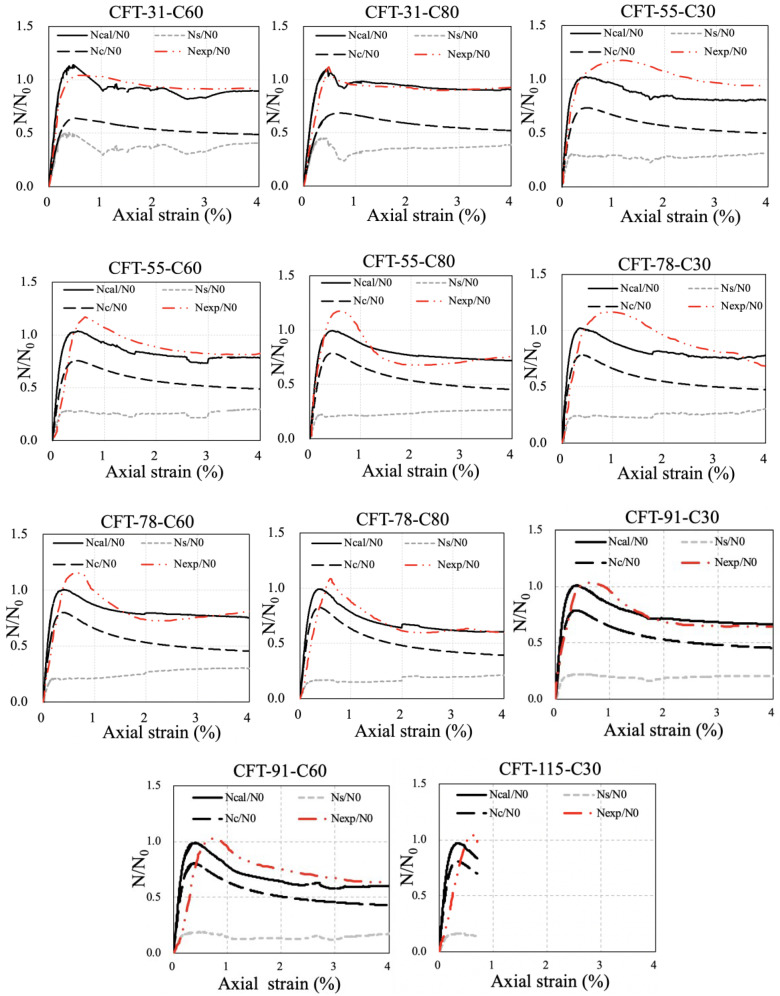
Comparison of experimental curves and calculated results by proposed method.

To verify the reliability of the proposed elastoplastic analytical method, superposition results of the load–strain curves of the steel tube and core concrete were compared with the experimental curves. Figure 15 shows the comparisons of the non-dimensional experimental curves *N*_exp_/*N*_0_-*ε* and calculated results *N_cal_*/*N*_0_-*ε* of the specimens in this paper. *N*_exp_ is the measured axial load of the specimens. *N_cal_* is the calculated axial load of the specimens, which can be expressed by Equation (24). *N*_0_ is the nominal squash load provided in Equation (25).

(24) $$N_{cal}=N_{c}+N_{s}=A_{c}f_{c}+A_{s}\psi_{TM-ave}f_{s}$$

(25) $$N_{0}=A_{c}f_{cu,kcy}+A_{s}f_{sy}$$

where *N_s_* is the axial load of the steel tube calculated by the proposed method, *N_c_* is the axial load of the core concrete calculated by the confined concrete model proposed by Sakino–Sun [24], *f_c_* is the axial stress of the confined concrete model corresponding to any axial strain *ε_c_*, and *A_c_* and *A_s_* are the cross-section area of the core concrete and steel tube respectively.

The comparisons indicate that the calculated results are generally in satisfactory consistency with the experimental results, although the ultimate strength of some specimens is underestimated by the proposed method due to the calculation deviation. As shown in Figure 15, the experimental stiffness is smaller than the calculated one (e.g., CFT-91-C60, CFT-115-C30). The reason for this phenomenon is that the steel end plate as shown in Figure 1 might not be ideally flat as expected due to the welding and assembly, so at the initial loading stage, the loading surface of servo-controlled hydraulic machine had partial gaps with the steel end plate, while the calculated one did not consider the gap. Furthermore, to exhibit the load sharing pattern of the steel tube and core concrete, curves of *N_s_*/*N*_0_-*ε* and *N_c_*/*N*_0_-*ε* are also presented. This indicates that the infilled concrete shared more axial loading comparing with steel tube.

## 7. Conclusions

Due to the difficulty in obtaining the stress–strain relationship of steel tube of CFT columns, this paper proposed an analytical method to obtain the full-range stress–strain curves of the steel tube of the specimens based on the deformation theory of plasticity and experiments conducted on 11 CFT stub columns under uniaxial compression to investigate the mechanical behavior of the steel tube infilled with concrete. The load–strain curve of CFT and strain development of steel tube were measured and presented. The following conclusions can be drawn by the experimental and analytical results.

(1) The proposed method provides an effective way to analyze the measure strain history to obtain the experimental stress–strain curve of the steel tube of CFT considering biaxial stress state and local buckling, which is critical to comprehend the elastoplastic behavior of the steel tube interacted with the core concrete.
(2) Comparisons of the stress–strain curves of the steel tube and CHS indicated that the infilled concrete indeed helpfully improved the residual stress of the steel tube, but it would cause an earlier strength degradation of the steel tube. This feature is suggested to be considered in finite simulation work on CFT columns.
(3) Different from the specimens of Class 2 and Class 4, local buckling of the specimens of Class 5 was prior to the ultimate strength, which indicated that the influence of local buckling was greater on the columns with thinner-walled steel tube. To reduce the local buckling effect on the behavior of CFT columns, the thin-walled steel tube is suggested to be used carefully.
(4) Crushing of concrete in shear failure and local buckling of the steel tube were observed in all the specimens. The shear failed concrete caused secondary local buckling of the steel tube, which dominated buckle deformation of the steel tube with increasing axial compression.
(5) Higher strength concrete of specimens showed earlier shear failure due to less ductility. With increasing concrete strength and ratio of *D*/*t*, the strength degradation of the columns was steeper.

## 8. Future Work

The analytical results indicated that proposed method is an effective and simple approach to obtain the experimental stress–strain curve of the steel tube of CFT. Based on the obtained experimental stress–strain curve of the steel tube of CFT, the mathematic axial stress–strain model of steel tube of CFT is anticipated to be built to consider the biaxial stress state and local buckling, which are not clearly considered or just considered based on simulation results in current constitutive model of steel tube infilled with concrete. These works are in progress based on our previous proposed model of CHS [42], which showed that the mathematic axial stress–strain model of steel tube of CFT in progress had higher accuracy than existing models, and will be reported in the near future.

## Figures and Tables

**Table 1:** Details of the specimens.

Specimens	*D* (mm)	*t* (mm)	*H*(mm)	*f_cu,k_*(MPa)	*f_cu,kcy_*(MPa)	*f_sy_*(MPa)	*f_su_*(MPa)	*A* (%)	*Es* (GPa)	*α*	Type
CFT-31-C60	219	7.06	659	70	60	429	663	25	205	0.06	Class2
CFT-31-C80	219	7.06	659	93	81	429	663	25	205	0.06	Class 2
CFT-55-C30	273	5.00	816	58	50	312	452	35	202	0.08	Class 4
CFT-55-C60	273	5.00	816	70	60	312	452	35	202	0.08	Class 4
CFT-55-C80	273	5.00	816	93	81	312	452	35	202	0.08	Class 4
CFT-78-C30	273	3.50	813	58	50	305	444	36	205	0.12	Class 5
CFT-78-C60	273	3.50	813	70	60	305	444	36	205	0.12	Class 5
CFT-78-C80	273	3.50	818	93	81	305	444	36	205	0.12	Class 5
CFT-93-C30	325	3.57	971	58	50	329	453	33	206	0.15	Class 5
CFT-93-C60	325	3.57	971	70	60	329	453	33	206	0.15	Class 5
CFT-115-C30	376	3.28	1125	58	50	305	435	37	181	0.17	Class 5

## Data Availability

Not applicable.

## Appendix A

The expressions of the CHS model are listed below. *ε*_s_ is any given compressive strain and the *f*_s_ is the stress corresponding to the *ε*_s_. *N* is the round coefficient determining the ascending portion. The ascending branch can be defined by Equation (A1) to Equation (A6). Peak stress and peak strain can be defined by Equation (A7) to Equation (A9). The descending branch can be defined by Equation (A10) to Equation (A13).

(A1) $$f_{s}=E_{s}\varepsilon_{s}\left(Q+\frac{1-Q}{{\left(1+{\left|\varepsilon_{s}/\varepsilon_{ch}\right|}^{N}\right)}^{1/N}}\right)$$

(A2) $$S=\frac{1}{0.82+1.22\alpha }$$

(A3) $$\varepsilon_{sy}=\frac{f_{sy}}{E_{s}}$$

(A4) $$\varepsilon_{sm}=\frac{0.25}{\alpha^{1.3}}\varepsilon_{sy}$$

(A5) $$Q=0.03\varepsilon_{sm}^{-0.63},\;\varepsilon_{sm}\;in\;\%$$

(A6) $$\varepsilon_{ch}=\frac{Sf_{sy}-QE_{s}\varepsilon_{sm}}{\left(1-Q\right)E_{s}}$$

(A7) $$f_{sm}=E_{s}\varepsilon_{sm}\left(Q+\frac{1-Q}{{\left(1+{\left|\varepsilon_{sm}/\varepsilon_{ch}\right|}^{N}\right)}^{1/N}}\right)$$

(A8) $$\varepsilon_{sm}=\frac{0.25f_{sy}}{E_{s}\alpha^{1.3}}$$

(A9) N=6

(A10) $$f_{s}=\frac{A}{B+\varepsilon_{s}}$$

(A11) $$A=\frac{f_{res}f_{sm}\left(0.04-\varepsilon_{sm}\right)}{f_{sm}-f_{res}}$$

(A12) $$B=\frac{0.04f_{res}-f_{sm}\varepsilon_{sm}}{f_{sm}-f_{res}}$$

(A13) $$f_{res}=f_{sm}\left(0.12+0.23\varepsilon_{sm}\right),\;\varepsilon_{sm}\;in\;\%$$
